# Intra-Abdominal Pressure as a Marker of Enteral Nutrition Intolerance in Critically Ill Patients. The PIANE Study

**DOI:** 10.3390/nu11112616

**Published:** 2019-11-01

**Authors:** M Luisa Bordejé, Juan C. Montejo, M Lidón Mateu, Manuel Solera, Jose A. Acosta, Mar Juan, Francisco García-Córdoba, Miguel A. García-Martínez, Rosa Gastaldo, PIANE STUDY GROUP SPAIN

**Affiliations:** 1 ICU, Hospital Universitario Germans Trias i Pujol, Carretera del Canyet s/n, 08916 Badalona, Spain; 2 ICU, Hospital Universitario 12 de Octubre, Glorieta de Málaga s/n, 28041 Madrid, Spain; 3 ICU, Hospital General Universitario de Castellón, Avda. Benicassim s/n, 12004 Castellon, Spain; mateuli@hotmail.com; 4 ICU, Hospital San Francisco de Borja Pg. les Germanies 71, 46702 Gandía, Valencia, Spain; manuuci@hotmail.com; 5 ICU, Hospital General Unversitario de Alicante, C/Maestro Alonso 109, 03010 Alicante, Spain; acostesc@gmail.com; 6 ICU, Hospital Clínico Universitario de Valencia, Av. de Blasco Ibáñez, 17, 46010 Valencia, Spain; mar604@hotmail.com; 7 ICU, Hospital Universitario Los Arcos del Mar Menor, Paraje Torre Octavio s/n, 30739 Pozo Aledo—San Javier, Murcia, Spain; pagacor@gmail.com; 8 ICU, Hospital Universitario de Torrevieja. Carretera CV-95 s/n, 03186 Torrevieja, Alicante, Spain; garciamartinez.ma@gmail.com; 9 ICU, Hospital de Manacor Alcudia s/n, 07500 Manacor, Islas Baleares, Spain; manacorcali@gmail.com

**Keywords:** enteral nutrition, enteral nutrition intolerance, gastrointestinal complications, intensive care unit, intra-abdominal pressure

## Abstract

To determine whether elevated intra-abdominal pressure (IAP) is associated with a higher rate of enteral nutrition-related gastrointestinal (GI) complications; to assess the value of IAP as a predictor of enteral nutrition (EN) intolerance. Intensive Care Unit (ICU) patients on mechanical ventilation requiring at least 5 days of EN were recruited for a prospective, observational, non-interventional, multicenter study. EN was performed and GI complications were managed with an established protocol. IAP was determined via a urinary catheter. Patients who developed any GI complications were considered as presenting EN intolerance. Variables related to EN, IAP and GI complications were monitored daily. Statistical analysis compared patients without GI complications (group A) vs. GI complications (group B). 247 patients were recruited from 28 participating ICUs (group A: 119, group B: 128). No differences between groups were recorded. Patients in group B (*p* < 0.001) spent more days on EN (8.1 ± 8.4 vs. 18.1 ± 13.7), on mechanical ventilation (8.0 ± 7.7 vs. 19.3 ± 14.9) and in the ICU (12.3 ± 11.4 vs. 24.8 ± 17.5). IAP prior to the GI complication was (14.3 ± 3.1 vs. 15.8 ± 4.8) (*p* < 0.003). The best IAP value identified for EN intolerance was 14 mmHg but it had low sensitivity and specificity. Although a higher IAP was associated with EN intolerance, IAP alone did not emerge as a good predictor of EN intolerance in critically ill patients.

## 1. Introduction

Enteral nutrition (EN) is the preferred route for artificial nutrition in critically ill patients [1]. EN appears to protect the structure and function of the digestive tract, limiting bacterial translocation [2], and it is associated with fewer, and less severe, complications than parenteral nutrition [3,4]. Indeed, early EN (initiated within 24 h of patient admission) has been associated with reductions in the incidence of infectious complications, hospital stay, and mortality [5]. However, EN is associated with a high incidence of related gastrointestinal (GI) complications in critically ill patients [6,7].

In clinical practice, EN intolerance is considered in critically ill patients who present signs and symptoms such as vomiting, regurgitation, abdominal distension, diarrhea and constipation. Our study group has previously worked on defining and managing GI complications in Intensive Care Unit (ICU) patients [6]. To better define and manage gastric intolerance in these patients, we proposed an increased threshold (500 mL) for “normal” gastric residual volume (GRV) [8]. However, the value of GRV and the frequency of this measure for monitoring EN tolerance in critically ill patients is controversial [1,9,10].

In this study, we investigate the value of intra-abdominal pressure level (IAP) as a marker for anticipating, or predicting, the development of GI complications in these patients. Several authors have associated increased IAP values with respiratory, cardiovascular, renal and GI dysfunctions [11,12,13,14,15,16,17,18,19]. Increased levels of pro-inflammatory cytokines may be involved in organ dysfunction after elevated IAP values [20,21,22]. Given the effects of IAP on GI function, some authors propose that IAP values should be included in scores of GI failure or bowel dysfunction [23,24].

As a working hypothesis, we anticipated that an increased level of IAP may be associated with EN intolerance in critically ill patients. We also assessed whether IAP values could predict EN intolerance in these patients.

## 2. Materials and Methods

An observational, non-interventional, prospective multicenter study was conducted in 28 adult Intensive Care Units (ICUs) in Spain over a four-month period. Adult patients admitted to ICU were enrolled if they were on mechanical ventilation, and required Enteral Nutrition (EN) at least for an anticipated period of five days. EN was applied via a nasogastric tube. Patients fed through a duodenal or jejunal tube were not included. The correct position of the feeding tube was radiographically confirmed before diet infusion.

Patients who presented characteristics that might interfere with IAP measurement, such as ascites, bladder surgery or neurogenic bladder, were excluded from the study.

Participating investigators at each center calculated patients’ nutritional requirements and indicated the type of enteral formula diet. Investigators were instructed to follow the recommendations of the Spanish Nutritional and Metabolic Working Group to calculate each patient’s requirements [25]. The type of diet and caloric intake, between 25–30 Kcal/kg/d was left to the criterion of each researcher. The first day started the diet at 50% of the target calculated and the second day, the goal was to reach 100%.

Metoclopramide (10 mg IV every 8 h) was administered during the first three days of EN. A previously established protocol was used for EN administration and for the definition and management of GI complications. Patients were examined daily to detect the presence of gastrointestinal complications. EN intolerance was considered if the patients developed any GI complication during their ICU stay. In case of intolerance, the amount of diet administered was reduced according to the specific algorithm for each complication. The IAP value was not used to modify EN.

The following GI complications were defined according to criteria published by our group [6,8]: a) Abdominal distension: presence of a change in abdominal exploration, and increased abdominal girth diameter and abdominal cavity with respect to the previous exploration; b) High GRV: recovered gastric volume equal to or greater than 500 mL in each GRV evaluation. GRV was measured at 6 h intervals during the 1st EN day, every 8 h the 2nd EN day, and on a daily basis after the 3rd day of tolerated EN. GRV measurement was continued every 6 h while patients were EN intolerant. No maximum daily GRV was defined; c) Vomiting: enteral formula ejected through the mouth; d) Diet regurgitation: enteral formula found in oral or nasal cavities with or without exteriorization; e) Diarrhea: five or more liquid stools in a 24-h period or an estimated stool volume equal to or greater than 2000 mL/day; and f) Constipation: Absence of stool production after 7 days of EN, or absence of deposition for three consecutive days from the first week of EN administration [6,7].

Pulmonary aspiration of the enteral formula was also assessed. Aspiration was diagnosed when enteral formula was visualized in the routine tracheal aspirate. Pneumonia was defined according to CDC/NHSN criteria [26]. No microbiological confirmation was required for a diagnosis of pneumonia.

Patients were followed until the end of EN in the ICU, ICU discharge, or death in ICU. Data collection was limited to a maximum of 28 ICU days. Patients were withdrawn from the study if EN was stopped for more than 12 consecutive hours for any reason.

IAP was measured by attaching an Abdo-Pressure^®^ System (CONVATEC^®^) to the urinary bladder catheter. Measurements were performed every 6 h at end-expiration with patients in the supine position after ensuring the absence of abdominal muscle contractions, and zeroing the transducer at the level of the midaxillary line. IAP was expressed in mmHg.

### 2.1. Variables

Demographic variables and the admission diagnosis were collected upon ICU admission. Severity scores (APACHE II and SOFA scores) for the first 24 h were also recorded. The following EN variables were recorded: time from admission to EN, calculated energy requirements, enteral formula, GI complications, EN duration, and cause of EN withdrawal. The volume ratio (VR) was estimated as a measure of the efficacy of daily nutritional administration and calculated as follows: VR (%) = (volume administered/volume prescribed) × 100.

IAP was measured every six hours during the study. For statistical purposes, we defined *maximum daily IAP* as the highest IAP recorded each day. Pre-GI complication IAP was defined as the IAP value closest in time to a subsequent complication.

Outcome variables investigated were days on mechanical ventilation, length of stay in ICU, first 24 h SOFA score, day 5 SOFA score, final SOFA score (SOFA at the end of EN), and patient’s final outcome.

Data were prospectively collected online using a web-based clinical report form (CRF) completed by the physician responsible for patients at each center. Discrepancies and transcription errors were discussed with the main investigators (LB, JCM) and clarified before submitting each CRF for data validation and statistical analysis.

### 2.2. Statistical Methods

Collected data were analyzed by an independent institution (Servei d’Estadística de la Universitat Autònoma de Barcelona, Barcelona, Spain) using the software package SPSS version 19.0 for Windows.

Categorical variables were expressed as number and percentage of patients by response category. Continuous variables were provided as means and standard deviations (SD) or medians and IQR (interquartile range), according to the Kolmogorov-Smirnov test which was used to test normal distribution. Categorical variables were compared using the Chi-Square test. Continuous variables with normal distribution were compared using the Student’s t-test (for two categories) and the ANOVA test (for more than two categories). When continuous variables were not normally distributed, the statistical test used was the Mann–Whitney U test for two categories or the Wilcoxon test for more than two categories.

To determine the best IAP value for predicting EN intolerance (diagnostic test evaluation), the receiver operating curve (ROC) curve was performed. Assuming an α error = 5% and an β error = 10%, the required sample size was estimated at 220 cases. Significance was set at *p* < 0.05.

#### Ethics Approval and Consent to Participate

The study protocol was approved by the institutional review boards at the two coordinating centers: Comité Etico de Investigación Clínica del Hospital Germans Trias i Pujol (ref number: EO-11-078) and “Comité Etico de Investigación Clínica del Hospital Universitario 12 de Octubre (ref number: 11/248) and was applicable to participating centers. Informed consent was obtained from patients or their legal surrogates at all participating centers. The study was performed in accordance with established ethical standards.

## 3. Results

A total of 257 patients were recruited. Each participating center included 8.8 ± 3.0 (mean ± SD) patients (range 2–14). Ten patients were excluded from the final analysis due to incomplete IAP or EN data. Group A (patients without GI complications) comprised 119 patients and group B (patients with GI complications) 128.

No differences between the groups were recorded in age, sex distribution, diagnosis, APACHE II score, SOFA score and time from admission to EN (Table 1).

In total, 2494 EN days were monitored. High-protein diets were used in 35.7% of the EN days, glycemic-control diets in 23.4%, ARDS diets in 18.1% and pharmaconutrition diets in 10.5%. The type of diet did not determine the occurrence of further complications. The mean daily diet volume administered was similar in the two groups. Volume ratio was higher in patients without GI complications. More patients in group A were weaned from EN and switched to an oral diet (Table 1).

Diarrhea was the main GI complication in group B patients (19%), followed by constipation (Table 2).

Patients with GI complications (group B) had more EN days, mechanical ventilation days and length of stay in ICU than patients without GI complications (group A). Nevertheless, ICU mortality was similar in the two groups (Table 1).

IAP values differed between groups (Table 3). Mean daily IAP was similar, but maximum daily IAP was higher in group B patients. For group B patients, mean IAP value before GI complications was higher: 15.8 ± 4.8 mmHg (mean ± SD).

**Figure 1:**
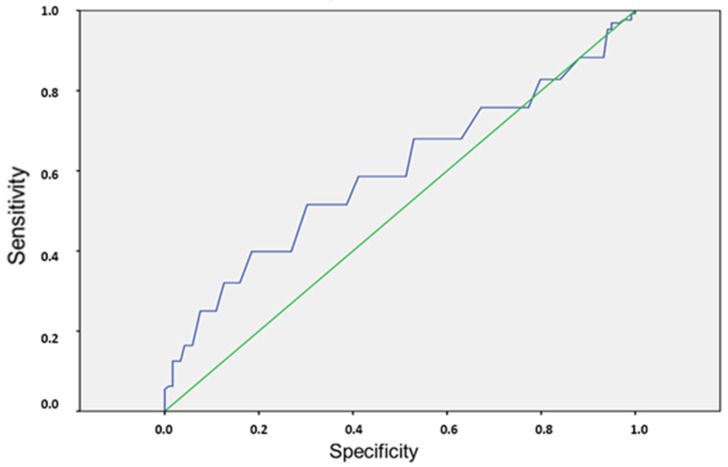
Receiver operating curve (ROC) of intra-abdominal pressure (IAP) recorded immediately before the first gastrointestinal complication in patients with gastrointestinal complications (group B) or the mean daily IAP recorded in patients without gastrointestinal complications (group A). Area under the curve = 0.595, indicating a low diagnostic power of IAP to predict gastrointestinal complications.

**Figure 2:**
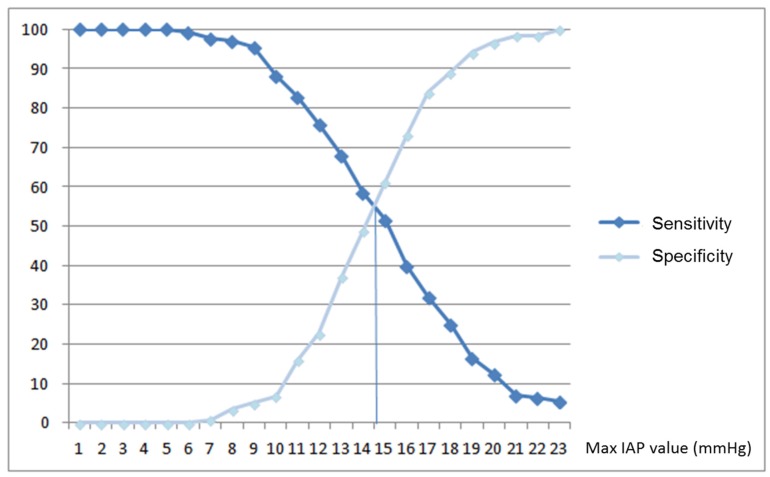
Sensitivity and specificity of maximum intraabdominal pressure (IAP) in predicting enteral nutrition intolerance. The best IAP cut-off of 14 mmHg showed a sensitivity of 58.6% and a specificity of 48.7%.

A receiver operating curve (ROC) was constructed considering mean daily IAP values for group A patients and pre-GI complication IAP values for group B. The area under the curve was 0.595, indicating a low diagnostic power of IAP to predict the occurrence of GI complications (Figure 1). An IAP value of 14 mmHg was identified in sensitivity versus specificity curves as the best cut-off to predict GI complications, but it had low sensitivity (58.6%) and low specificity (48.7%) (Figure 2).

## 4. Discussion

The results of this prospective, multicenter study indicate that patients who were not able to tolerate EN showed higher IAP values.

The relationship between IAP and EN intolerance has been examined by other authors. Combining enteral diet tolerance and IAP values, Reintam et al. (23) developed a GI failure score with prognostic value for ICU mortality. In that study the authors did not assess the possible link between IAP and EN tolerance, though in a later trial [27], they reported no such relationship. However, they did note that patients with both high IAP and signs of diet intolerance had worse outcomes. The complex relationship between IAP and enteral feeding tolerance has been explored in several studies designed to clarify the terminology related to these two factors and their management [28,29].

Bejarano et al. [30] assessed the relationship between baseline IAP (before starting EN) and EN tolerance. According to their findings, APACHE II scores combined with IAP served to predict a patient’s EN tolerance. In their predictive model, patients with higher APACHE II scores showed intolerance at a lower IAP value, while patients with lower APACHE scores needed a higher IAP value. In our study, the group with intolerance did not present a higher APACHE value, probably due to their higher degree of complexity, given that our goal was not to measure tolerance at the beginning of EN but to measure intolerance during ICU stay. Also, the criteria for intolerance were not the same. In the study by Bejarano et al. [30] a GRV > 200 mL was already considered as intolerance; patients in their study with an IAP of 14 mmHg before EN started had a high risk of EN intolerance if their APACHE II was above 14. In our study, this IAP value of 14 mmHg was found to be the best cut-off point for predicting EN intolerance. However, given its poor sensitivity and specificity, we do not recommend its use in clinical practice. Certain differences between the study populations in these two studies should be noted; while the Bejarano et al. [30] sample comprised mainly surgical or trauma patients, in our study, medical diagnoses were predominant. Additionally, patients receiving EN through a jejunal tube were included in the Bejarano et al. [30] study but not in ours. The influence of patient diagnosis or enteral feeding tube position on IAP and its effect on EN tolerance is speculative, and cannot be addressed with the data available at present, but it is a very interesting possibility.

Hill et al. [31] also examined the effect of elevated IAP on GI function. Despite the small number of patients in their study, these authors observed that a higher IAP was associated with worse GI function, EN intolerance, a longer hospital stay and higher mortality. So far, based on both published data and our own findings, we have been unable to establish an IAP threshold for predicting EN intolerance in critically ill patients. The possibility that IAP may be a valuable predictor of EN intolerance is an attractive hypothesis, but it requires further investigation.

Interestingly, our participants presented a mean IAP of 14.8 mmHg (Table 3). According to the Intra-abdominal Hypertension consensus conference [12], this value classifies our patients as having “moderate intra-abdominal hypertension.” Our data also indicate that IAP values for our patients were clearly above the value defined at that same meeting as being “normal” for critically ill patients. Other authors have also reported a higher IAP than the consensus value for this patient population [14]. We measured IAP daily every six hours. When we analyzed them, we realized that there were important variations between daily IAP determinations. We justify it by the fact that IAP measurements can be influenced by many factors such as abdominal surgery, obesity, mechanical ventilation, the determination itself, and others [32,33,34], so we thought mean daily IAP was more reliable than maximum daily IAP. In view of these reports, we feel there is a need to revise the current definitions of IAP and abdominal compartment syndrome, and the relevance for critically ill patients.

The main limitations of our study are the subjectivity of the definition of GI symptoms and the heterogeneity of these patients, and the ICUs involved. A mean IAP of 14 mmHg is a low degree of intra-abdominal hypertension. It is known that the higher the IAP value, the greater the abdominal complications, and presumably also the adverse impact on outcomes; therefore, it would be advisable to expand the sample so as to increase the number of patients with higher IAP values.

In previous work, we observed that the main complication related to EN in critically ill patients was an increased gastric residual volume [6]. However, by raising the accepted threshold of GRV to 500 mL [8], we noted that other complications such as diarrhea and constipation were more frequent than high GRV. The incidence of diarrhea was higher than that reported in other publications but equal to that obtained in previous studies conducted by our group, in which the same definitions were used for GI complications [8].

The presence of GI complications had prognostic consequences. Affected patients showed worse outcome variables, such as a longer duration of mechanical ventilation and hospital stay, though mortality rates were unaffected. The detrimental effects of GI complications during EN have also been described by other authors [35]. However, it is still unknown whether the worse outcomes observed are a direct consequence of the patients’ GI complications or whether they reflect the action of other factors. Further work is needed to clarify this issue.

The present study is the first prospective, multicenter trial designed to determine whether there is an IAP value able to predict EN intolerance in critically ill, mechanically ventilated patients. One of the strengths of our study is the fact that it was conducted by a group of investigators with wide experience in nutrition therapy in the critically ill and related research areas. Enteral nutrition was protocolized, as were the definition and management of EN-related GI complications. In addition, IAP was measured at the participating centers using the same procedure and equipment. Despite our efforts, however, we could not confirm the existence of a suitable IAP value for predicting EN intolerance. Future studies focusing on more specific sets of critical patients and using new methods to measure IAP [36] may clarify or modify our results.

## 5. Conclusions

In this study, we tested the hypothesis that increased values of intraabdominal pressure (IAP) could be used as a marker for EN intolerance in critically ill patients. Our results indicate that IAP values are increased in patients with EN intolerance. Nevertheless, we did not find a cut-off point for IAP able to predict EN intolerance. The effect of IAP on EN tolerance in critically ill patients should be investigated in more detail.

## Figures and Tables

**Table 1:** Demographic, enteral nutrition and outcome variables recorded in critically ill, mechanically ventilated patients without (group A) and with gastrointestinal complications (group B).

	Overall	GROUP A (*n* = 119)	GROUP B (*n* = 128)	*p*
Age (years) (mean ± SD)	62.0 ± 14.7	62.5 ± 15.6	61.6 ± 13.9	0.64
Sex distribution (%):				0.37
Males	63.6%	66.4%	60.9%	
Females	36.4%	33.6%	39.1%	
Admission diagnosis (% of patients):				0.42
Medical	79.8%	83.1%	76.5%	
Surgical	5.7%	5.0%	6.3%	
Trauma	8.9%	5.9%	11.8%	
APACHE II (first 24 h) (mean ± SD)	21.4 ± 7.8	22.1 ± 8.6	20.8 ± 6.8	0.187
SOFA on admission (mean ± SD)	7.5 ± 3.2	7.6 ± 3.0	7.5 ± 3.5	0.78
Admission to EN (hours) (mean ± SD)	30.6 ± 23.5	30.2 ± 23.0	30.9 ± 24.1	0.82
median (P25; 75)	24 (3; 99)	23 (3; 96)	24 (4; 99)	
EN volume administered (mL/day)				
(mean ± SD)	1107.6 ± 396.1	1062.4 ± 375.8	1149.6 ± 411.1	0.08
EN volume ratio * (mean ± SD)	86.9 ± 22.2%	88.6 ± 20.6%	86.1 ± 22.8%	0.009
Transition to oral diet (% of patients)	42.5%	52.9%	32.8%	<0.002
EN days (mean ± SD)	13.3 ± 12.5	8.1 ± 8.4	18.1 ± 13.7	<0.001
Mechanical ventilation days				
(mean ± SD)	13.8 ± 13.2	8.0 ± 7.7	19.3 ± 14.9	<0.001
ICU days (mean ± SD)	18.8 ± 16.1	12.3 ± 11.4	24.8 ± 17.5	<0.001
ICU death	52 (21.1%)	24 (20.2%)	28 (22.0%)	0.757

APACHE: Acute Physiology and Chronic Health Evaluation, SOFA: Sepsis-Related Organ Failure Assessment, EN: enteral nutrition, * EN volume ratio (%) = (EN volume administered/EN volume prescribed) × 100, ICU: Intensive Care Unit.

**Table 2:** Rate of gastrointestinal complications.

Complication (%)	*n*	All Patients (*n* = 247)	GROUP B (*n* = 128)
Diarrhea	47	19.0%	36.7%
Constipation	43	17.4%	33.6%
High gastric residual volume	40	16.2%	31.25%
Abdominal distension	28	11.3%	21.8%
Vomiting	24	9.7%	18.7%
Diet regurgitation	16	6.5%	12.5%
Aspiration	2	0.8%	1.5%

**Table 3:** Intra-abdominal pressure (IAP) variables recorded in critically ill, mechanically ventilated patients without (group A) and with gastrointestinal complications (group B).

		Overall	GROUP A (*n* = 119)	GROUP B (*n* = 128)	*P*
**Daily IAP**	Mean ± SD	14.8 ± 4	14.8 ± 3.7	14.8 ± 4.1	0.801
**Maximum daily IAP**	Mean ± SD	18.1 ± 4.6	16.8 ± 4	19.4 ± 4.8	<0.001

## Abbreviations

ACS	Abdominal compartmental Syndrome
APACHE II	Acute Physiologic and chronic health evaluation II
ARDS	Acute Respiratory Distress Syndrome
EN	enteral nutrition
GI	gastrointestinal
GRV	gastric residual volume
IAP	intraabdominal pressure
ICU	Intensive Care Unit
SOFA	Sequential Organ Failure Assessment
